# A New Anæsthetic

**Published:** 1852-01

**Authors:** 


From the Dental News Letter.

### A NEW ANÆSTHETIC.

Messrs. Editors:—Permit me to call the attention of your readers to the following article, which appears in the March number of Sillman's Journal, descriptive of a *new anæsthetic*, and its *superior efficacy* in the *production* of *local narcotism*. In the event of the further confirmation of the properties assigned to the agent therein described, it will prove to be, as it is, so far as tested, evidently one of great power; indeed, superior to any which we have heretofore possessed, and will be of almost incalculable advantage and benefit in the treatment of disease and the performance of operations, and particularly appropriate to those included in the practice of dentistry, the application to which is the design, more especially, of the present notice. Its properties rendering it peculiarly adapted to the treatment of the dental pulp, and that exceedingly sensitive condition of the animal tissue so often exhibited in the decay of the teeth, and the effectual and painless removal of which, preparatory to the more final operation, presents obstacles of no mean importance, as those who have it to perform can well understand and appreciate; therefore, anything favorable to the successful removal of these difficulties should be considered of the highest importance, and, I hope will be acknowledged of sufficient, to *excuse this mode of directing attention* to a remedy so promising. Respectfully yours, GEO. J. ZIEGLER.

---

"*Anæsthetic Action.*—(L'Institut, No. 886.)—M. Aran has made experiments on the *anæsthetic action* of certain agents used as an *external application* to the *skin*, and has found that the *best material* for this purpose *is chlorated chlorohydric ether.* The sesquichlorid of carbon may also be used; but whilst the *ether operates effectually in a few minutes,* at least two hours are required to produce insensibility with the sesquichlorid. To produce the desired effect, from 15 to 30 drops of the pure chlorated chlorohydric ether suffice; they are put upon the part in pain, or upon a piece of linen cloth, which is to be immediately applied to this part, and the contact is maintained by a bandage, and *quickly the pain is relieved.* A pomatum of this ether may also be employed, consisting of four grammes to 20 of suet; or if of the sesquichlorid of carbon, four of this agent to 30 of suet; it may be used either with friction or without. *The insensibility is not simply cutaneous, for it gradually extends to the parts beneath.*

"*The chlorated chlorohydric ether is obtained by the action of chlorine on hydrochloric ether,* by which compounds containing chlorine in increasing proportions are formed, isomerous with the series of bicarburets of hydrogen, and identical with the same series in the density of the vapor for corresponding compounds. It is a colorless liquid, of an etherial aromatic odor, analogous to chloroform, and a sweetish and even peppery taste at times; hardly soluble in water, but wholly so in alcohol, sulphuric ether, and most of the fixed and volatile oils. It is without action upon paper of tournsol; is not inflammable; has a variable density and a variable point of ebullition, oscillating between 110° and 130° C., showing that the material is rather a mixture of several ethers than a simple substance. *All the chlorated chlorohydric ethers have the same anæsthetic properties,* and they cannot be separated completely from one another.—*Amer. Jour. of Sci. and Arts,* March, 1851.

